# Beyond the Immediate Impact: Burnout, Psychological Distress, and Workforce Retention Among Healthcare Workers One Year After the Türkiye Earthquakes

**DOI:** 10.3390/healthcare14121599

**Published:** 2026-06-06

**Authors:** Neslihan Cansel, Osman Kurt, Ayça Elçim Sahar Gürbüz, Merve Bulut, Şahide Nur İpek Melez, Burcu Kayhan Tetik

**Affiliations:** 1 Department of Psychiatry, Inonu University Faculty of Medicine, Malatya 44100, Türkiye; aelcimsahar@gmail.com; 2 Department of Public Health, Inonu University Faculty of Medicine, Malatya 44100, Türkiye; drkurtosman@gmail.com; 3 Department of Family Medicine, Inonu University Faculty of Medicine, Malatya 44100, Türkiye; mervebulut1182@gmail.com (M.B.); drburcukayhan@hotmail.com (B.K.T.); 4 Department of Internal Medicine, Gaziantep City Hospital, Gaziantep 27000, Türkiye

**Keywords:** earthquake, healthcare workers, burnout, post-traumatic stress disorder, anxiety, depression, disaster mental health

## Abstract

**Highlights:**

**What are the main findings?**

- One year after the Türkiye earthquakes, healthcare workers demonstrated high levels of depression, PTSD risk, and persistently low personal accomplishment despite lower emotional exhaustion and depersonalization.
- Disaster-related exposures and organizational factors, particularly job satisfaction, were associated with burnout dimensions, psychiatric symptoms, and intention to leave the profession.

**What are the implications of the main findings?**

- Psychological burden among healthcare workers may extend beyond the acute disaster phase, suggesting the need for longitudinal monitoring during recovery periods.
- Organizational strategies targeting workplace conditions and access to mental health support may play a key role in workforce retention and disaster preparedness.

**Abstract:**

**Background/Objectives:** This study aimed to evaluate burnout, psychological distress, and intention to quit among healthcare workers one year after the 6 February 2023 earthquakes, and to examine the relative contributions of disaster-related exposures and organizational factors using a hierarchical analytical approach. **Methods:** This cross-sectional study included 640 healthcare workers from a tertiary referral hospital in one of the provinces most severely affected by the earthquakes. Data were collected using validated instruments, including the Maslach Burnout Inventory, Hospital Anxiety and Depression Scale, Impact of Event Scale–Revised, and Intention to Quit Scale. Hierarchical multiple linear regression analyses were performed to evaluate factors associated with burnout dimensions, psychiatric symptoms, and intention to quit. **Results:** Clinically significant anxiety symptoms were observed in 32.5% of participants, depressive symptoms in 55.8%, and PTSD risk in 54.1%. Low personal accomplishment was the most prevalent burnout dimension (69.1%), while high emotional exhaustion and depersonalization were observed in 43.0% and 18.9% of participants, respectively. Workplace climate variables accounted for the largest increment in explained variance across all seven models. Low job satisfaction was the strongest and most consistent factor associated with adverse outcomes, with standardized coefficients ranging from β = +0.27 to +0.61. Non-close colleague relations were independently associated with higher burnout, anxiety, depression, and intention to quit scores, as well as lower personal accomplishment. Despite the high prevalence of psychological symptoms, post-earthquake psychiatric help-seeking was reported by only 6.2% of participants. **Conclusions:** One year after the earthquakes, healthcare workers continued to experience a substantial psychological burden. Although disaster-related exposures were associated with several adverse outcomes, organizational factors appeared to demonstrate more consistent associations with mental health indicators. These findings highlight the potential importance of improving modifiable workplace conditions to support psychological well-being and workforce sustainability in post-disaster healthcare systems.

## 1. Introduction

Earthquakes generate large-scale physical destruction and substantial loss of life while simultaneously imposing sustained pressure on healthcare systems [1,2]. Healthcare workers are often required to maintain uninterrupted service delivery under conditions characterized by increased patient demand, limited resources, and repeated exposure to traumatic events, placing them at elevated risk for adverse mental health outcomes such as burnout, depression, and post-traumatic stress disorder (PTSD) [3,4]. These psychological effects may influence not only individual well-being but also workforce stability and the sustainability of healthcare delivery in disaster-affected settings [5,6]. Burnout is characterized by emotional exhaustion, depersonalization, and reduced personal accomplishment, while anxiety, depression, and PTSD represent broader psychological responses to occupational and trauma-related stressors [7,8,9]. Previous studies suggest that these domains are interrelated and may collectively contribute to workforce attrition; however, the relative contribution of disaster-related exposures and organizational factors remains insufficiently understood.

Existing evidence indicates that psychological outcomes among healthcare workers after disasters are shaped not only by traumatic exposure but also by occupational and organizational conditions, including workload, professional relationships, and job satisfaction [10,11,12,13]. On 6 February 2023, two major earthquakes measuring Mw 7.7 and Mw 7.6 struck southeastern Türkiye, causing widespread destruction, displacement, and major disruption to healthcare services [14,15,16]. Healthcare workers in affected provinces were required to continue providing care despite being directly exposed to the disaster themselves. Although previous studies have reported increased psychological distress among healthcare workers following earthquakes, most available data originate from the acute post-disaster phase [17,18]. However, psychological responses may evolve over time as initial social support and crisis-related mobilization decline and prolonged occupational strain persists. Longitudinal evidence suggests that while PTSD symptoms may gradually decrease, burnout, anxiety, and depressive symptoms may persist during longer-term recovery periods [12,19,20].

Importantly, relatively few studies have simultaneously evaluated burnout, psychiatric symptoms, and intention to quit within a unified analytical framework incorporating demographic, disaster-related, and organizational determinants. In addition, the relative contribution of organizational factors compared with direct disaster exposure remains unclear in the medium-term post-disaster period. Addressing this gap, the present study aimed to evaluate burnout, anxiety, depression, PTSD symptoms, and intention to quit among healthcare workers one year after the February 6 earthquakes in one of the most severely affected provinces in Türkiye. Using a hierarchical modeling approach, the study further aimed to examine the relative contributions of demographic characteristics, disaster-related exposures, and organizational factors to psychological outcomes.

We hypothesized that (i) disaster-related exposures would be significantly associated with psychological distress and burnout, and (ii) organizational factors, particularly job satisfaction, would demonstrate stronger and more consistent associations with psychological outcomes and intention to quit after adjustment for demographic and exposure-related variables.

## 2. Materials and Methods

### 2.1. Study Design and Participants

This cross-sectional study was conducted among healthcare workers employed in different units at Turgut Özal Medical Center, a tertiary-level academic hospital and the largest referral center in the region. The hospital is located in Malatya, one of the provinces most severely affected by the 6 February 2023 Kahramanmaraş earthquakes, and continued to provide uninterrupted healthcare services throughout the post-disaster period.

Healthcare workers from all hospital departments were eligible to participate. Inclusion criteria included active employment as a healthcare worker at the institution during both the earthquake period and the study period, as well as residence in Malatya before and after the earthquakes. Participants were recruited using a convenience sampling approach, including healthcare workers available during the data collection period. Because convenience sampling was used, selection bias cannot be excluded and the representativeness of the sample may be limited. In addition, although approximate recruitment numbers were available, recruitment was conducted informally across multiple departments and work schedules; therefore, the exact denominator of all eligible healthcare workers who were approached could not be verified with certainty, and a precise response rate could not be calculated.

**Figure 1:**
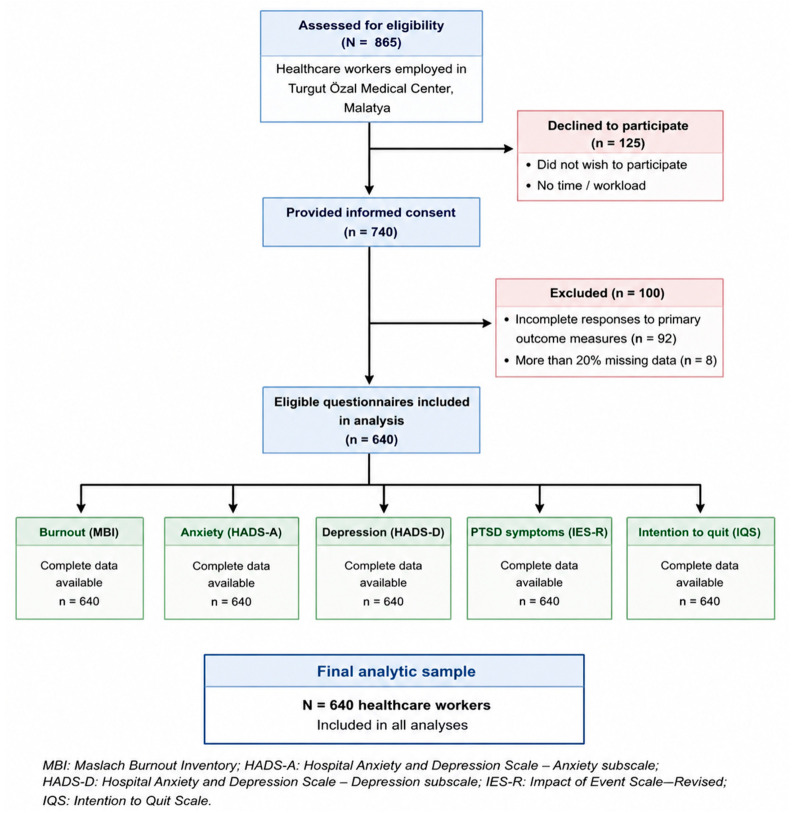
Flow Diagram of Participant Recruitment, Eligibility Assessment, and Final Inclusion in the Analysis.

Participation was voluntary and anonymous, and no financial compensation was provided. The study was conducted in accordance with the Declaration of Helsinki and was approved by the İnönü University Faculty of Medicine Non-Interventional Ethics Committee (Approval No: 2023/5398). Written informed consent was obtained from all participants. Participants with a prior psychiatric history were not excluded because the study aimed to evaluate psychological outcomes within a real-world healthcare workforce population. Instead, psychiatric history was included as a covariate in the regression analyses. Figure 1 presents the participant recruitment process, exclusions, and final analytic sample.

### 2.2. Data Collection Procedure

Data were collected between February and March 2024, corresponding to the first anniversary of the earthquakes. Printed questionnaire forms were distributed in person through face-to-face administration by trained research personnel. Participants received standardized information regarding the study aims, procedures, confidentiality, and voluntary participation, and written informed consent was obtained prior to enrollment.

Of the completed questionnaires, 100 were excluded because of incomplete responses in the primary outcome measures or excessive missing data, resulting in a final analytic sample of 640 participants. Questionnaires with incomplete responses in the primary outcome measures were excluded using a complete-case approach, and no data imputation procedures were performed. All measures were self-reported, which may introduce response bias. The questionnaire consisted of five sections: Sociodemographic, Occupational Characteristics, and Earthquake-Related Experiences (Section 1); the Maslach Burnout Inventory (MBI; Section 2); the Hospital Anxiety and Depression Scale (HADS; Section 3); the Impact of Event Scale–Revised (IES-R; Section 4); and the Intention to Quit Scale (Section 5).

### 2.3. Sample Size and Power Analysis

The sample size was determined using an a priori power analysis conducted with G*Power 3.1, based on parameters appropriate for hierarchical multiple linear regression (F-tests, fixed model, R^2^ deviation from zero). Consistent with a previous study [21], a moderate effect size of f^2^ = 0.15 (corresponding to R^2^ ≈ 0.13) was anticipated. With α = 0.05, power (1 − β) = 0.80, and 26 predictors, the minimum required sample size was estimated at 175 participants. The final sample of 640 substantially exceeded this threshold (post hoc power > 0.99 for all models).

### 2.4. Data Collection Instruments

The data form was developed by the research team based on a review of the relevant post-disaster literature and comprised four domains: (1) sociodemographic characteristics (age, sex, marital status, having children, educational level, and monthly income); (2) occupational characteristics (professional role, department of employment, shift work arrangement, length of employment, job satisfaction, and colleague relations); (3) personal health and lifestyle factors (psychiatric history, chronic disease, engagement in sports activity, hobbies and leisure pursuits, and prior trauma history); and (4) earthquake-related experiences (personal loss, housing damage, displacement, help-seeking behavior, and anxiety about future earthquakes).

The Maslach Burnout Inventory (MBI) was used to assess burnout across three subscales: emotional exhaustion (9 items), depersonalization (5 items), and personal accomplishment (8 items) [22]. The Turkish adaptation of the MBI employed a 5-point Likert scale ranging from 0 (never) to 4 (always), modified from the original 7-point format [23]. Burnout levels were categorized consistent with established scoring conventions: for EE, low 0–11, moderate 12–17, and high ≥ 18; for DP, low 0–5, moderate 6–9, and high ≥ 10; and for PA, low ≥ 26, moderate 22–25, and high 0–21 [24]. Cronbach’s α coefficients from the original Turkish adaptation were 0.83 for EE, 0.78 for DP, and 0.74 for PA; the corresponding values in the present sample were 0.913, 0.779, and 0.801.

The Hospital Anxiety and Depression Scale (HADS) was employed to assess the severity of anxiety and depression symptoms. This 14-item self-report instrument comprises two subscales: anxiety (HADS-A; 7 items) and depression (HADS-D; 7 items), each scored on a 4-point Likert scale ranging from 0 to 3, yielding subscale totals between 0 and 21 [25]. In the Turkish validation study, Cronbach’s α coefficients were 0.85 for the anxiety subscale and 0.77 for the depression subscale, with scores of ≥11 and ≥8 indicating clinically significant anxiety and depression, respectively [26]. In the present sample, the corresponding values were 0.826 and 0.765, respectively.

The Impact of Event Scale–Revised (IES-R) was employed to assess the psychological impact of a traumatic event [27]. This 22-item self-report measure evaluates three dimensions of post-traumatic stress symptoms—intrusion, avoidance, and hyperarousal—rated on a Likert-type scale ranging from 0 (“not at all”) to 4 (“extremely”). The Turkish validity and reliability study reported a Cronbach’s α of 0.937 for the total scale, with a total score of ≥33 proposed as a clinical cutoff for PTSD risk [28]. In the present sample, Cronbach’s α coefficients were 0.938 for the total scale, 0.915 for intrusion, 0.773 for avoidance, and 0.893 for hyperarousal.

The Intention to Quit Scale (IQS) was employed to assess employees’ intention to quit their current position [29]. This four-item instrument uses a 7-point Likert scale ranging from 1 (close to zero) to 7 (close to 100%), with higher scores indicating greater intention to quit. Cronbach’s α was 0.913 in the Turkish study and 0.835 in the present sample [30].

### 2.5. Statistical Analysis

Descriptive statistics were reported as mean ± standard deviation (SD) and median (minimum–maximum) for continuous variables, and as frequency and percentage for categorical variables. The normality of continuous variables was assessed using the Shapiro–Wilk test. Spearman correlation analysis was used to examine relationships among continuous outcome measures. Hierarchical multiple linear regression models were constructed to identify variables independently associated with burnout dimensions, psychiatric symptoms, and intention to quit. Predictor variables were entered in five theoretically derived blocks. The incremental contribution of each block was evaluated using the change in R^2^ (ΔR^2^) and the associated F-change statistic; overall model fit was assessed using the F-statistic and adjusted R^2^. Block 1 comprised demographic variables (age, sex, marital status, having children, educational level, income). Block 2 comprised structural occupational variables (professional role, department of employment, shift work arrangement, length of employment). Block 3 comprised personal vulnerability and resource variables (psychiatric history, chronic disease, sports activity, hobby and leisure engagement, prior trauma history). Block 4 comprised earthquake-related exposures (housing damage, loss of a close relative, city relocation). Block 5 comprised workplace climate variables (job satisfaction, colleague relations). For each final model, standardized (β) and unstandardized (B) regression coefficients, standard errors, 95% confidence intervals, and variance inflation factors (VIFs) are reported. Multicollinearity was evaluated using VIF, and values exceeding 5.0 were considered potentially problematic. Regression assumptions, including linearity, normality of residuals, homoscedasticity, and independence of residuals, were evaluated using residual plots, normal probability plots, and Durbin–Watson statistics. Statistical significance was set at *p* < 0.05 (two-tailed). All analyses were performed using IBM SPSS Statistics 26.0 (IBM Corp., Armonk, NY, USA).

## 3. Results

### 3.1. Participant Characteristics

The demographic and occupational characteristics of the participants are presented in Table 1. The mean age was 34.50 ± 8.36 years, and 54.1% of participants were female. Most participants were married (60.3%), and 50.5% had children. University-level education or above was reported by 82.7% of the sample. Physicians (29.4%), nurses (28.9%), and allied health personnel (30.3%) constituted the majority of occupational groups. Most participants were employed in internal medicine departments (58.9%) and had worked for three years or longer (63.0%). Moderate job satisfaction was reported by 59.8% of participants, while 58.6% described their colleague relations as close.

### 3.2. Distribution of Earthquake-Related Experiences

Earthquake-related exposures and post-disaster conditions are summarized in Table 2. Direct exposure to the earthquakes was reported by 97.7% of participants, and 39.7% reported prior trauma history. Temporary relocation to another city occurred in 39.8% of participants, while 69.5% continued to work in shift-based schedules after the disaster. Housing damage was reported as mild in 57.0% and moderate-to-severe in 29.1% of participants. Loss of a close relative was reported by 13.4%. Post-earthquake psychiatric help-seeking was reported by only 6.2% of participants. Anxiety about future earthquakes was present in 85.2% of participants, and 45.0% reported plans to relocate because of the disaster.

### 3.3. Scale Scores and Prevalence of Psychological Symptoms

Descriptive statistics for all outcome measures are presented in Table 3. Clinically significant anxiety symptoms were identified in 32.5% of participants, depressive symptoms in 55.8%, and PTSD risk in 54.1%. Among burnout dimensions, high emotional exhaustion was observed in 43.0% of participants and high depersonalization in 18.9%, whereas low personal accomplishment was observed in 69.1%. The mean intention to quit score was 4.56 ± 3.01.

### 3.4. Correlations Between Scale Scores and Continuous Variables

Spearman correlation analyses demonstrated significant correlations among most outcome measures (Table 4). Emotional exhaustion showed positive correlations with depersonalization (r = 0.658), anxiety (r = 0.499), depression (r = 0.414), PTSD (r = 0.446), and intention to quit (r = 0.519) (all *p* < 0.001). Depersonalization was positively correlated with anxiety, depression, PTSD, and intention to quit (all *p* < 0.001). Personal accomplishment demonstrated inverse correlations with anxiety (r = −0.199), depression (r = −0.190), and intention to quit (r = −0.150) (all *p* < 0.001), but was not significantly associated with PTSD (*p* = 0.339). Anxiety, depression, and PTSD scores were also significantly intercorrelated (all *p* < 0.001).

### 3.5. Factors Associated with Burnout Dimensions, Anxiety, Depression, PTSD, and Intention to Quit

The results of the hierarchical multiple linear regression analyses are summarized in Table 5, while detailed block-level statistics are provided in Supplementary Table S1. Final models explained 35.2% of the variance in emotional exhaustion, 27.0% in intention to quit, 21.7% in depersonalization, 19.4% in anxiety, 16.0% in depression, 14.4% in PTSD, and 7.3% in personal accomplishment.

Workplace climate variables (Block 5) demonstrated the largest contribution to explained variance across all models. Low job satisfaction was associated with higher emotional exhaustion (β = +0.61), depersonalization (β = +0.33), anxiety (β = +0.29), depression (β = +0.26), PTSD (β = +0.27), and intention to quit (β = +0.50), and with lower personal accomplishment (β = −0.11) (all *p* < 0.05). Non-close colleague relations were associated with higher emotional exhaustion, depersonalization, anxiety, depression, and intention to quit scores (all *p* < 0.05).

Among occupational variables, working in surgical units was associated with higher emotional exhaustion (β = +0.13), depersonalization (β = +0.15), anxiety (β = +0.22), depression (β = +0.14), and PTSD scores (β = +0.18) (all *p* < 0.05). Physician status demonstrated an inverse association with intention to quit (β = −0.19, *p* = 0.004), while nurse status was associated with higher emotional exhaustion and anxiety scores.

Among demographic variables, male gender was associated with higher depersonalization and intention to quit scores, but lower anxiety and PTSD scores. Older age was inversely associated with depersonalization and positively associated with personal accomplishment. University-level education was associated with lower anxiety, depression, and depersonalization scores.

Among personal vulnerability and resource variables, psychiatric history was associated with higher emotional exhaustion (β = +0.07, *p* = 0.038). Sports activity was associated with lower anxiety and depression scores and higher personal accomplishment, while engagement in hobbies was associated with lower depression and higher personal accomplishment scores.

Among earthquake-related exposures, mild housing damage was associated with higher anxiety (β = +0.15) and PTSD scores (β = +0.17), while severe housing damage was associated with higher depression scores (β = +0.12). Loss of a close relative was associated with greater intention to quit (β = +0.07, *p* = 0.049).

## 4. Discussion

This study demonstrated that healthcare workers in the affected region continued to experience a substantial psychological burden one year after the 2023 earthquakes. Clinically significant anxiety symptoms were observed in approximately one-third of participants, whereas depressive symptoms and PTSD risk were present in more than half of the sample. Among burnout dimensions, low personal accomplishment was the most prevalent finding, followed by emotional exhaustion and depersonalization. These findings provide insight into the medium-term psychological effects of disasters on healthcare workers.

Previous studies evaluating healthcare workers during the early post-disaster period have primarily emphasized acute stress responses characterized by emotional exhaustion and depersonalization associated with increased workload and emotional demands [31,32]. Although direct comparisons are limited because of methodological differences across studies, the present findings indicate that reduced personal accomplishment and psychiatric symptom burden remained highly prevalent one year after the disaster. This pattern may reflect the persistence of occupational strain during the recovery phase, when the initial mobilization period and external social support mechanisms gradually diminish. However, because of the cross-sectional design, these findings should be interpreted as associative rather than temporal observations. An important aspect of the present study was the simultaneous evaluation of burnout, psychiatric symptoms, and intention to quit within a hierarchical analytical framework. Emotional exhaustion demonstrated the highest explained variance among the regression models, whereas personal accomplishment demonstrated the lowest. Workplace climate variables contributed the largest increase in explained variance across all outcomes, particularly for emotional exhaustion and intention to quit. Low job satisfaction demonstrated consistent associations with all adverse psychological outcomes, while poor colleague relations were associated with higher burnout, anxiety, depression, and intention to quit scores. These findings are consistent with previous literature emphasizing the role of organizational climate and workplace conditions in healthcare worker mental health [33,34,35,36]. Occupational characteristics also demonstrated significant associations across several outcomes. Working in surgical units was associated with higher emotional exhaustion, depersonalization, anxiety, depression, and PTSD scores, while nurse status was associated with higher emotional exhaustion and anxiety scores. These findings are generally consistent with previous studies reporting increased psychological strain among frontline healthcare workers exposed to intensive clinical workloads and emotionally demanding environments [4,33,34]. Physician status demonstrated an inverse association with intention to quit, which may reflect differences in occupational roles or professional commitment; however, the mechanisms underlying this association remain uncertain. Earthquake-related exposure variables demonstrated more selective associations. Housing damage was associated with higher anxiety, depression, and PTSD scores, whereas loss of a close relative was associated with greater intention to quit. Previous studies have similarly reported that cumulative disaster exposure and ongoing environmental stressors may contribute to sustained psychological distress following major disasters [37,38]. Persistent housing instability and bereavement may represent continuing stressors extending beyond the acute disaster period.

Gender-related differences were also observed in several models. Male gender was associated with higher depersonalization and intention to quit scores, whereas female gender was associated with higher PTSD scores. Similar sex-related differences in burnout and trauma-related symptoms have been reported previously [39,40,41]. However, the underlying mechanisms remain incompletely understood, and these findings should be interpreted cautiously given the multifactorial nature of psychological responses following disasters. Older age was associated with lower depersonalization and higher personal accomplishment, while having children was inversely associated with depersonalization. These findings are broadly consistent with studies suggesting that greater professional experience and social support structures may be associated with more favorable occupational coping patterns [42]. Psychiatric history demonstrated an association with higher emotional exhaustion scores, consistent with previous evidence indicating that pre-existing mental health vulnerability may be associated with increased susceptibility to occupational stress [43]. In contrast, prior trauma history was positively associated with personal accomplishment. Similar findings have been discussed within post-traumatic growth frameworks, suggesting that previous coping experiences may influence professional self-perception during subsequent stressful events [44]. Educational attainment and income demonstrated differing associations across outcomes. Higher educational level was associated with lower anxiety, depression, and depersonalization scores, whereas higher income was associated with greater depersonalization and intention to quit. Previous studies have reported inconsistent findings regarding socioeconomic indicators and occupational mental health outcomes [45,46]. These associations may reflect differences in occupational expectations, responsibilities, or career opportunities; however, causal interpretations cannot be established from the present findings. The observed associations between burnout, psychiatric symptoms, and intention to quit support the interconnected nature of these outcomes. Previous studies have similarly suggested that occupational burnout and psychological distress may coexist and collectively contribute to turnover intentions among healthcare workers [47,48,49]. In disaster-affected healthcare systems, such patterns may have implications for workforce continuity and institutional resilience. Sports activity and engagement in hobbies demonstrated associations with more favorable psychological outcomes in several models. Similar observations have been reported in disaster and pandemic settings, where participation in structured physical activity and meaningful leisure activities was associated with lower psychological distress [50,51]. These findings may support the potential relevance of recovery-oriented and lifestyle-related approaches in post-disaster mental health support strategies.

### 4.1. Clinical and Theoretical Implications

The findings highlight the importance of targeted strategies to support healthcare worker mental health during disaster recovery periods. Frontline healthcare workers, individuals experiencing displacement or loss of a close relative, and those reporting low job satisfaction demonstrated higher levels of psychological burden across several outcomes. The consistent associations observed between workplace-related variables and psychological outcomes may indicate the potential relevance of organizational interventions targeting job satisfaction, team dynamics, institutional communication, and workload distribution. In addition, engagement in hobbies, leisure activities, and structured non-work routines was associated with more favorable psychological outcomes in several models. A notable finding of the present study was the discrepancy between the high prevalence of psychological symptoms and the very low rate of post-earthquake psychiatric help-seeking. This pattern may reflect barriers such as stigma, limited time, workload intensity, restricted access to mental health services, or normalization of psychological distress among healthcare workers. These findings may support the importance of proactive and institution-based mental health support strategies in post-disaster healthcare settings.

### 4.2. Limitations

Several limitations should be considered when interpreting the findings of this study. First, the cross-sectional design precludes causal inference and limits the ability to establish temporal relationships between predictors and outcomes; therefore, the observed associations should not be interpreted as evidence of causation or directionality. Second, all data were collected using self-report instruments, raising the possibility of response bias, including social desirability bias and underreporting of psychological symptoms. The use of a single data collection method at one time point may also have increased the risk of common method bias. Third, the study employed a convenience sampling strategy, and a precise response rate could not be calculated. Therefore, selection and non-response bias cannot be excluded, and the representativeness of the sample may be limited. In addition, healthcare workers who left the province or resigned from their positions after the earthquakes may not have been represented in the study sample, potentially leading to underrepresentation of individuals most severely affected by the disaster. Fourth, although a broad range of variables was included in the hierarchical regression models, residual confounding from unmeasured factors—such as workload intensity, institutional support, leadership quality, and individual coping styles—may have influenced the findings. Fifth, the absence of pre-earthquake baseline data limits the ability to determine whether the observed psychological burden reflects post-disaster changes or pre-existing conditions. In addition, the study design did not allow detailed evaluation of the reasons underlying the low rate of mental health help-seeking despite the high prevalence of psychological symptoms. Finally, multiple statistical models were constructed across several related outcomes without formal correction for multiple comparisons. Although the consistency of findings across models may support the robustness of the results, the possibility of type I error cannot be completely excluded.

## 5. Conclusions

One year after the earthquakes, healthcare workers continued to demonstrate a substantial psychological burden, including high levels of burnout, depressive symptoms, and PTSD risk. Although disaster-related exposures were associated with several adverse psychological outcomes, organizational factors—particularly job satisfaction and colleague relations—demonstrated more consistent associations across the hierarchical models. These findings suggest that workplace-related conditions may play an important role in shaping psychological outcomes during the post-disaster recovery period. From a clinical and organizational perspective, interventions targeting institutional support, workplace climate, and recovery-oriented behaviors may contribute to supporting healthcare worker well-being and workforce sustainability in disaster-affected healthcare systems.

## Figures and Tables

**Table 1:** Demographic and occupational characteristics of participants.

Variable	*n*	%
Age (years)	Mean 34.50 ± 8.36	Median 33 (17–58)
Female gender	346	54.1
Married	386	60.3
Has children	323	50.5
University education or above	529	82.7
Occupation		
Physician	188	29.4
Nurse	185	28.9
Allied health personnel	194	30.3
Administrative staff/technical/other	73	11.4
Department of employment		
Internal medicine	377	58.9
Surgical unit	178	27.8
Administrative/Technical/Other	85	13.3
Length of employment		
Less than 1 year	91	14.2
1–3 years	146	22.8
3 years or more	403	63.0
Monthly income		
≤Minimum wage	14	2.2
Minimum wage—cost of living	283	44.2
≥Cost-of-living benchmark	343	53.6
Psychiatric history	39	6.1
Chronic disease	109	17.0
Sports activity	114	17.8
Hobby/interests	470	73.4
Job satisfaction		
High	139	21.7
Moderate	383	59.8
Low	118	18.4
Colleague relations		
Close	375	58.6
Not close	265	41.4

Binary variables are presented as the positive/primary category only. SD = Standard Deviation. Income categories were based on the national minimum wage and cost-of-living benchmark in Türkiye at the time of data collection.

**Table 2:** Distribution of earthquake-related experiences and post-disaster conditions (*N* = 640).

Variable	*n*	%
Direct exposure to the earthquakes	625	97.7
Prior trauma history	254	39.7
Temporary relocation to another city	255	39.8
Housing damage		
No damage	89	13.9
Mild damage	365	57.0
Moderate to severe damage	186	29.1
Shift-based work arrangement	445	69.5
Loss of a close relative	86	13.4
Psychiatric help after earthquake	40	6.2
Anxiety about future earthquake	545	85.2
Plans to relocate due to earthquake	288	45.0

Binary variables are presented as the positive/primary category only.

**Table 3:** Distribution of scale scores and prevalence of psychological symptoms.

Scale	Subscale	Mean ± SD	Median (Min–Max)	*n* (%)
MBI	EE	15.98 ± 8.41	16.0 (0–36)	Low: 204 (31.9) Moderate: 161 (25.2) High: 275 (43.0)
	DP	5.62 ± 4.31	5.0 (0–20)	Low: 349 (54.5) Moderate: 170 (26.6) High: 121 (18.9)
	PA	18.81 ± 5.56	19.0 (0–32)	Low: 442 (69.1) Moderate: 136 (21.2) High: 62 (9.7)
	MBI Total	34.79 ± 13.21	35.0 (3–76)	—
HADS	HADS-A	8.57 ± 4.41	8.0 (0–21)	Normal: 432 (67.5) Abnormal: 208 (32.5)
	HADS-D	8.05 ± 3.93	8.0 (0–21)	Normal: 283 (44.2) Abnormal: 357 (55.8)
	HADS Total	16.63 ± 7.60	17.0 (0–42)	—
IESR	Intrusion	13.98 ± 8.33	13.0 (0–46)	—
	Avoidance	12.66 ± 5.90	12.0 (0–32)	—
	Hyperarousal	9.35 ± 6.32	9.0 (0–24)	—
	IES-R Total	35.99 ± 18.36	35.0 (0–88)	PTSD absent: 294 (45.9) PTSD present: 346 (54.1)
IQS	Total score	4.56 ± 3.01	4.0 (1–28)	—

MBI: Maslach Burnout Inventory; EE, Emotional Exhaustion; DP, Depersonalization; HADS, Hospital Anxiety and Depression Scale; HADS-A, Anxiety, HADS-D, Depression IES-R, Impact of Event Scale–Revised; IQS, Intent to Quit Scale.

**Table 4:** Spearman correlation coefficients between scale scores and continuous variables.

Variable	1	2	3	4	5	6	7
1. EE	—	0.658 ***	−0.111 **	0.499 ***	0.414 ***	0.446 ***	0.519 ***
2. DP		—	−0.186 ***	0.325 ***	0.250 ***	0.308 ***	0.391 ***
3. PA			—	−0.199 ***	−0.190 ***	−0.038	−0.150 ***
4. HADS-A				—	0.636 ***	0.671 ***	0.289 ***
5. HADS-D					—	0.541 ***	0.285 ***
6. PTSD						—	0.319 ***
7. IQS							—

EE = Emotional Exhaustion; DP = Depersonalization; PA = Personal Accomplishment; HADS-A = Hospital Anxiety and Depression Scale–Anxiety; HADS-D = Hospital Anxiety and Depression Scale–Depression; IES-R = Impact of Event Scale–Revised; IQS = Intention to Quit Scale. ** *p* < 0.01 *** *p* < 0.001.

**Table 5:** Hierarchical Regression Models for Burnout, Psychiatric Symptoms, and Intention to Quit Among Healthcare Workers.

Variable	EE	DP	PA	ANX	DEP	PTSD	ITQ
Adjusted R^2^	0.352	0.217	0.073	0.194	0.160	0.144	0.270
Block 1: Demographic variables							
Age	—	−0.15 **	+0.14 **	—	—	—	—
Male gender	—	+0.14 ***	—	−0.13 ***	—	−0.10 *	+0.09 *
Has children	—	−0.13 *	—	—	—	—	—
University+	—	−0.10 *	—	−0.11 *	−0.13 **	—	—
Income high	—	+0.15 **	—	—	—	—	+0.14 **
Block 2: Structural occupational variables							
Physician	+0.12 *	—	—	—	—	—	−0.19 **
Nurse	+0.15 **	—	—	+0.14 *	—	—	—
Internal medicine	—	+0.14 *	−0.12 *	—	—	—	—
Surgical unit	+0.13 *	+0.15 **	—	+0.22 ***	+0.14 *	+0.18 **	—
Block 3: Personal vulnerability/resources							
Psychiatric history	+0.07 *	—	—	—	—	—	—
Sports activity	—	—	+0.10 *	−0.09 *	−0.08 *	—	—
Hobby/interests	—	—	+0.13 **	—	−0.12 **	—	—
Prior trauma history	—	—	+0.09 *	—	—	—	—
Block 4: Earthquake-related exposures							
Housing dmg: mild	—	—	—	+0.15 **	—	+0.17 **	—
Housing dmg: severe	—	—	—	—	+0.12 *	—	—
Loss of relative	—	—	—	—	—	—	+0.07 *
Block 5: Workplace climate variables							
Job sat: moderate	+0.42 ***	+0.25 ***	−0.13 *	+0.20 ***	+0.18 ***	+0.18 ***	+0.28 ***
Job sat: low	+0.61 ***	+0.33 ***	−0.11 *	+0.29 ***	+0.26 ***	+0.27 ***	+0.50 ***
Colleague not-close	+0.11 **	+0.09 *	−0.09 *	+0.08 *	+0.13 ***	—	+0.12 ***

Note. Values represent standardized regression coefficients (β). Only variables with at least one statistically significant association (*p* < 0.05) are shown; non-significant associations indicated by —. Variables entered in all models but non-significant across all outcomes are omitted from display. Block-level model statistics are provided in Supplementary Table S1. EE, Emotional Exhaustion; DP, Depersonalization; PA, Personal Accomplishment; ANX, Anxiety (HADS-A); DEP, Depression (HADS-D); PTSD, Post-Traumatic Stress; ITQ, Intention to Quit. Max VIF = 3.72. * *p* < 0.05 ** *p* < 0.01 *** *p* < 0.001.

## Data Availability

The datasets generated and/or analyzed during the current study are not publicly available due to privacy and ethical restrictions but are available from the corresponding author upon reasonable request.

## Supplementary Materials

The following supporting information can be downloaded at: https://www.mdpi.com/article/10.3390/healthcare14121599/s1, Table S1: Block-Level Summary of Hierarchical Multiple Linear Regression Models (*N* = 640).

## Abbreviations

HCWs	Healthcare workers
MBI	Maslach Burnout Inventory
EE	Emotional Exhaustion
DP	Depersonalization
PA	Personal Accomplishment
HADS	Hospital Anxiety and Depression Scale
HADS-A	Hospital Anxiety and Depression Scale–Anxiety subscale
HADS-D	Hospital Anxiety and Depression Scale–Depression subscale
IES-R	Impact of Event Scale–Revised
PTSD	Post-traumatic stress disorder
IQS	Intention to Quit Scale
SD	Standard deviation
