# Supplementary material for: Beyond the Immediate Impact: Burnout, Psychological Distress, and Workforce Retention Among Healthcare Workers One Year After the Türkiye Earthquakes
Source: Healthcare (Basel). 2026 Jun 6;14(12):1599. doi: 10.3390/healthcare14121599 (PMC13299621; doi:10.3390/healthcare14121599)
Supplement: Supplementary file 1 [file healthcare-14-01599-s001.zip › healthcare-4273500-supplementary.pdf]

Supplementary Table S1. Block-Level Summary of Hierarchical Multiple Linear Regression Models (N = 640)

Outcome (Adj R^2)	Block	R^2	ΔR^2	F-change	df	p
**Emotional Exhaustion (Adj R^2 = 0.352)**	Block 1: Demographic variables	0.032	**0.032**	3.53	6, 633	**0.002**
	Block 2: Structural occupational variables	0.085	**0.052**	4.47	8, 625	**<.001**
	Block 3: Personal vulnerability/resources	0.119	**0.034**	4.77	5, 620	**<.001**
	Block 4: Earthquake-related exposures	0.122	0.004	0.65	4, 616	0.628
	Block 5: Workplace climate variables	0.379	**0.256**	84.27	3, 613	**<.001**
	**Final model: F(26, 613) = 14.37, p < .001 | Adjusted R^2 = 0.352**					
**Depersonalization (Adj R^2 = 0.217)**	Block 1: Demographic variables	0.106	**0.106**	12.48	6, 633	**<.001**
	Block 2: Structural occupational variables	0.138	**0.032**	2.88	8, 625	**0.004**
	Block 3: Personal vulnerability/resources	0.152	0.014	2.06	5, 620	0.069
	Block 4: Earthquake-related exposures	0.162	0.011	1.94	4, 616	0.103
	Block 5: Workplace climate variables	0.249	**0.086**	23.52	3, 613	**<.001**
	**Final model: F(26, 613) = 7.80, p < .001 | Adjusted R^2 = 0.217**					
**Personal Accomplishment (Adj R^2 = 0.073)**	Block 1: Demographic variables	0.029	**0.029**	3.17	6, 633	**0.005**
	Block 2: Structural occupational variables	0.046	0.017	1.40	8, 625	0.195
	Block 3: Personal vulnerability/resources	0.085	**0.039**	5.25	5, 620	**<.001**
	Block 4: Earthquake-related exposures	0.087	0.002	0.41	4, 616	0.800
	Block 5: Workplace climate variables	0.111	**0.023**	5.33	3, 613	**0.001**
	**Final model: F(26, 613) = 2.93, p < .001 | Adjusted R^2 = 0.073**					
**Anxiety (Adj R^2 = 0.194)**	Block 1: Demographic variables	0.031	**0.031**	3.42	6, 633	**0.002**
	Block 2: Structural occupational variables	0.094	**0.062**	5.38	8, 625	**<.001**
	Block 3: Personal vulnerability/resources	0.142	**0.048**	6.90	5, 620	**<.001**
	Block 4: Earthquake-related exposures	0.162	**0.021**	3.83	4, 616	**0.004**
	Block 5: Workplace climate variables	0.227	**0.065**	17.11	3, 613	**<.001**
	**Final model: F(26, 613) = 6.93, p < .001 | Adjusted R^2 = 0.194**					
**Depression (Adj R^2 = 0.160)**	Block 1: Demographic variables	0.018	0.018	1.93	6, 633	0.073
	Block 2: Structural occupational variables	0.062	**0.044**	3.65	8, 625	**<.001**
	Block 3: Personal vulnerability/resources	0.111	**0.050**	6.92	5, 620	**<.001**
	Block 4: Earthquake-related exposures	0.125	0.013	2.35	4, 616	0.053
	Block 5: Workplace climate variables	0.194	**0.069**	17.57	3, 613	**<.001**
	**Final model: F(26, 613) = 5.68, p < .001 | Adjusted R^2 = 0.160**					
**PTSD (Adj R^2 = 0.144)**	Block 1: Demographic variables	0.024	**0.024**	2.58	6, 633	**0.018**
	Block 2: Structural occupational variables	0.071	**0.048**	4.00	8, 625	**<.001**
	Block 3: Personal vulnerability/resources	0.099	**0.028**	3.80	5, 620	**0.002**
	Block 4: Earthquake-related exposures	0.125	**0.026**	4.65	4, 616	**0.001**

**Outcome (Adj R^2)**	**Block**	**R^2**	**ΔR^2**	**F-change**	**df**	**p**
	Block 5: Workplace climate variables	0.178	**0.053**	13.17	3, 613	**<.001**
	**Final model: F(26, 613) = 5.12, p < .001 | Adjusted R^2 = 0.144**					
**Intention to Quit (Adj R^2 = 0.270)**	Block 1: Demographic variables	0.029	**0.029**	3.17	6, 633	**0.005**
	Block 2: Structural occupational variables	0.088	**0.059**	5.02	8, 625	**<.001**
	Block 3: Personal vulnerability/resources	0.105	**0.017**	2.38	5, 620	**0.038**
	Block 4: Earthquake-related exposures	0.118	0.013	2.30	4, 616	0.057
	Block 5: Workplace climate variables	0.300	**0.182**	53.01	3, 613	**<.001**
	**Final model: F(26, 613) = 10.09, p < .001 | Adjusted R^2 = 0.270**					

R^2 = cumulative R^2 at each block entry; ΔR^2 = increment in R^2 attributable to the block; F-change = F-statistic for the R^2 increment; df = degrees of freedom (predictors added, residual); p = significance of the F-change. Bold ΔR^2 and p values indicate statistically significant block contributions ($p < .05$). Block 1: age, sex, marital status, having children, education, income. Block 2: profession (physician, nurse, allied HP; ref: administrative), department (internal medicine, surgical unit; ref: administrative/other), shift work, employment duration. Block 3: psychiatric history, chronic disease, sports activity, hobby/interests, prior trauma history. Block 4: housing damage mild, housing damage severe (ref: no damage), loss of a close relative, city relocation. Block 5: job satisfaction (moderate, low; ref: high), colleague relations (not close; ref: close).
